# C-754-01. Burn Injury Drives Biphasic Macrophage Immune Reprogramming Through Locus-Specific Epigenetic Remodeling

**DOI:** 10.1093/jbcr/irag033.029

**Published:** 2026-04-06

**Authors:** Hans Kim, Robert Maile, Aidan Higgs, Marie Gauthier, Denise A Hernandez, Jason Brant, Rhonda Bacher, Shannon M Wallet, Philip Efron, Michael P Kladde

**Affiliations:** University of Florida, Gainesville, FL; University of Florida, Gainesville, FL; National Institutes of Health, Washington, DC; University of Florida, Gainesville, FL; University of Florida, Gainesville, FL; University of Florida, Gainesville, FL; University of Florida, Gainesville, FL; University of Florida, Gainesville, FL; University of Florida, Gainesville, FL; University of Florida, Gainesville, FL

## Abstract

**Introduction:**

Severe burn injury is followed by immune dysfunction marked by early hyperinflammation and later immunosuppression. The underlying epigenetic mechanisms remain incompletely understood. We hypothesized that burn injury induces coordinated chromatin remodeling in macrophages that governs this biphasic immune phenotype.

**Methods:**

Female C57BL/6 mice (n = 6) underwent 20% TBSA full-thickness scald injury or sham procedure. Splenic F4/80^+^ macrophages were isolated on days 2, 9, and 14 post-injury. Cytokine secretion was measured following TLR2 (peptidoglycan) or TLR4 (LPS) stimulation. Targeted transcriptomic profiling of >1300 immune/metabolic genes was performed by nanoString. Epigenetic remodeling of 180 immune loci was assessed using single-molecule Methyltransferase accessibility protocol for individual templates combined with flap-enabled next-generation capture (MAPit-FENGC) to simultaneously measure DNA methylation and chromatin accessibility.

**Results:**

Macrophages from burn mice demonstrated a biphasic trajectory. At day 2, TLR-stimulated IL-6, MCP-1, and TNFα secretion were increased versus sham (*p*<.05). By day 14, MCP-1 and TNFα responses were suppressed, while IL-10 secretion increased (*p*<.05). Transcriptomic profiling confirmed dynamic regulation of Il10, Socs3, and cell-cycle genes, with persistent repression of Nfkb1, Traf6, and Stat3. MAPit-FENGC revealed early chromatin accessibility gains at proinflammatory loci (Cxcl15, Ccl7) and progressive repression of Stat3, Tgfb1, and Nfkb1 promoters. Nucleosome repositioning at the Il10 promoter preceded transcriptional upregulation, suggesting epigenetic priming for tolerance.

**Conclusions:**

Burn injury induces temporally coordinated transcriptomic and epigenetic remodeling in macrophages, shifting from hyperinflammation to immune tolerance. Locus-specific chromatin remodeling underlies this transition, highlighting mechanistic drivers of post-burn immune paralysis.

**Applicability of Research to Practice:**

These findings identify macrophage epigenetic reprogramming as a targetable mechanism of burn-induced immune dysfunction. Therapeutic interventions that recalibrate macrophage chromatin states may restore balanced immunity and reduce infection risk in burn survivors.

**Funding for the study:**

Supported by NIH NIGMS.

